# Habitat occupancy of sloth bear *Melursus ursinus* in Chitwan National Park, Nepal

**DOI:** 10.1002/ece3.8699

**Published:** 2022-03-06

**Authors:** Rajan Prasad Paudel, Rabin Kadariya, Babu Ram Lamichhane, Naresh Subedi, Mariko Sashika, Michito Shimozuru, Toshio Tsubota

**Affiliations:** ^1^ Laboratory of Wildlife Biology and Medicine Department of Environmental Veterinary Science Graduate School of Veterinary Medicine Hokkaido University Sapporo Japan; ^2^ National Trust for Nature Conservation Lalitpur Nepal

**Keywords:** Chitwan National Park, habitat use, Nepal, occupancy, sloth bear, wildlife conservation

## Abstract

Mammals have experienced a massive decline in their populations and geographic ranges worldwide. The sloth bear, *Melursus ursinus* (Shaw, 1791), is one of many species facing conservation threats. Despite being endangered in Nepal, decades of inattention to the situation have hindered their conservation and management. We assessed the distribution and patterns of habitat use by sloth bears in Chitwan National Park (CNP), Nepal. We conducted sign surveys from March to June, 2020, in 4 × 4 km grids (*n* = 45). We collected detection/non‐detection data along a 4‐km trail that was divided into 20 continuous segments of 200 m each. We obtained environmental, ecological, and anthropogenic covariates to understand determinants of sloth bear habitat occupancy. The data were analyzed using the single‐species single‐season occupancy method, with a spatially correlated detection. Using repeated observations, these models accounted for the imperfect detectability of the species to provide robust estimates of habitat occupancy. The model‐averaged occupancy estimate for the sloth bear was 69% and the detection probability was 0.25. The probability of habitat occupancy by sloth bears increased with the presence of termites and fruits and in rugged, dry, open, undisturbed habitats. Our results indicate that the sloth bear is elusive, functionally unique, and widespread in CNP. Future conservation interventions and action plans aimed at sloth bear management must adequately consider their habitat requirements.

## Graphical abstract

The current distribution and determinants of habitat use by the sloth bear in a protected area from Nepal are clarified using an occupancy method.

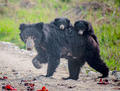

## 1 INTRODUCTION

**FIGURE 1:**
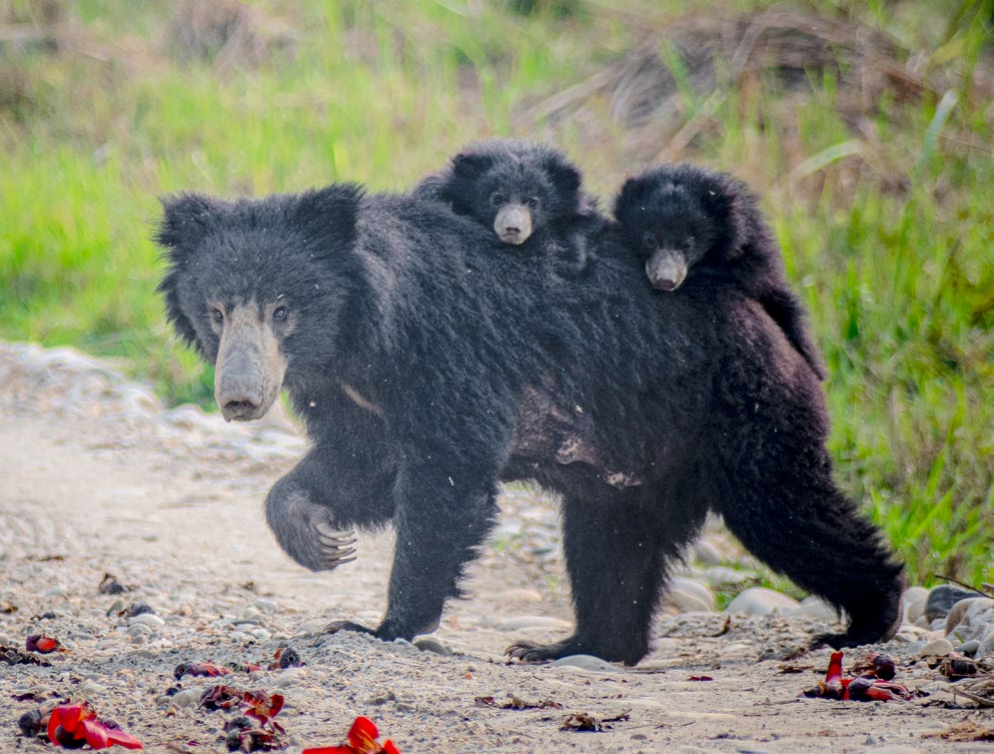
Sloth bear (*Melursus ursinus*) female with cubs photographed in its natural habitat at Chitwan National Park, Nepal. Photo credit: Arjun Tamang

The sloth bear *Melursus ursinus* (Shaw, 1791; Figure 1) is an endemic mammal of the Indian subcontinent that occurs in a wide range of habitats, including dry or moist forest, savannah, scrublands, and grasslands (Dharaiya et al., 2016; Garshelis et al., 1998). However, their populations have declined by almost 50% over the last three decades and the species is categorized as “vulnerable” in IUCN Red List of Threatened Species (Dharaiya et al., 2016). Sloth bears have been extirpated from Bangladesh (Islam et al., 2013) and possibly Bhutan (Dharaiya et al., 2016; Garshelis et al., 1998). They were once present along a continuous strip of forest and grasslands in southern Nepal until the 1950s when the expansion of human settlement and agriculture confined them primarily to a few protected areas (Amin et al., 2018; Jnawali et al., 2011). Information on determinants and patterns of habitat use are critical for setting conservation priorities and site‐specific management actions. A range of ecological and anthropogenic factors drive occupancy and habitat use by sloth bears in India (Babu et al., 2015; Das et al., 2014; Puri et al., 2015; Ramesh et al., 2012; Srivathsa et al., 2018) and Sri Lanka (Ratnayeke, Van Manen, Pieris, et al., 2007). The distribution, habitat use, population, and conservation ecology of the sympatric co‐predators, the tiger and leopard, are well documented in Nepal (Barber‐Meyer et al., 2013; Carter et al., 2012; Karki et al., 2015; Lamichhane et al., 2019; Pokheral & Wegge, 2019; Smith, 1984; Subedi, Lamichhane, et al., 2021; Thapa et al., 2021). However, such information for sloth bears is limited (Garshelis et al., 1998, 1999; Joshi et al., 1995, 1997, 1999; Lamichhane et al., 2016; Laurie & Seidensticker, 1977) and comparable habitat occupancy estimates are not available (Seidensticker et al., 2011). This gap in information has hindered management practices and the formulation of a conclusive view of the species’ current conservation status.

Species distribution and habitat use are primarily determined by the availability and spatial variation of food resources and the extent of natural and anthropogenic threats (Ceballos & Ehrlich, 2002; Schipper et al., 2008). Unlike other carnivores, bears exhibit a series of morphological specializations for their diet (Sacco & Valkenburgh, 2004). Sloth bears are specially adapted for a myrmecophagous diet (Joshi et al., 1997, 1999). The composition of the diet varies with the temporal and spatial availability of the food resources, particularly termites and fruits (Bargali et al., 2004; Baskaran et al., 2015; Joshi et al., 1997; Khanal & Thapa, 2014; Laurie & Seidensticker, 1977; Mewada, 2015; Mewada et al., 2019; Palei et al., 2014, 2020; Philip et al., 2021; Rather et al., 2020; Sukhadiya et al., 2013). In fruit‐rich areas, sloth bears play an important role in the dispersal of seed and regeneration of fruit plants, thereby aiding in the maintenance of forest structure and composition (Sreekumar & Balakrishnan, 2002). Reports of sloth bears from human‐dominated landscapes (Akhtar et al., 2004, 2007; Bargali et al., 2012; Puri et al., 2015) and the prevalence of human–sloth bear conflict in India (Bargali et al., 2005; Debata et al., 2017; Dhamorikar et al., 2017; Garcia et al., 2016; Ratnayeke et al., 2014; Sharp et al., 2020) and Nepal (Acharya et al., 2016; Lamichhane et al., 2018; Pokharel & Aryal, 2020; Silwal et al., 2017) suggest a high nexus between humans and sloth bears. They largely prefer habitats away from human disturbance (Babu et al., 2015; Ghimire & Thapa, 2014; Joshi et al., 1999; Ratnayeke, Van Manen, & Padmala, 2007; Ratnayeke, Van Manen, Pieris, et al., 2007). Removal of the individuals through poaching or live capture for use as “dancing bears” is not common, but maybe detrimental enough for a population that is already small, isolated, and threatened.

Chitwan National Park (CNP) is a key for wildlife habitat in Nepal. The highest density of sloth bears in Nepal is reported to occur in CNP (Garshelis et al., 1999). Translocation of this species from areas of high occupancy to suitable habitats outside CNP is recommended for its long‐term conservation (Jnawali et al., 2011). However, the lack of recent information on sloth bear distribution and habitat use patterns has hindered its conservation and management. Estimating their density and abundance is challenging due to their elusive nature and the difficulty in identifying individuals. The application of conventional methods such as camera traps, telemetry, and genetics can provide valuable information, but are logistically challenging and resource intensive. In contrast, occupancy methods account for imperfect detection to provide reliable ecological information when species research and monitoring are resource constrained or logistically challenging. This study was the first of its kind to use occupancy models to study the distribution and habitat use of sloth bears in Nepal. We established the current presence of sloth bears across the park and provided information on their distribution, habitat use, and associated covariates. The results will have far‐reaching implications for the research, management, and conservation of sloth bears in Nepal.

## 2 METHODOLOGY

### 2.1 Study area

**FIGURE 2:**
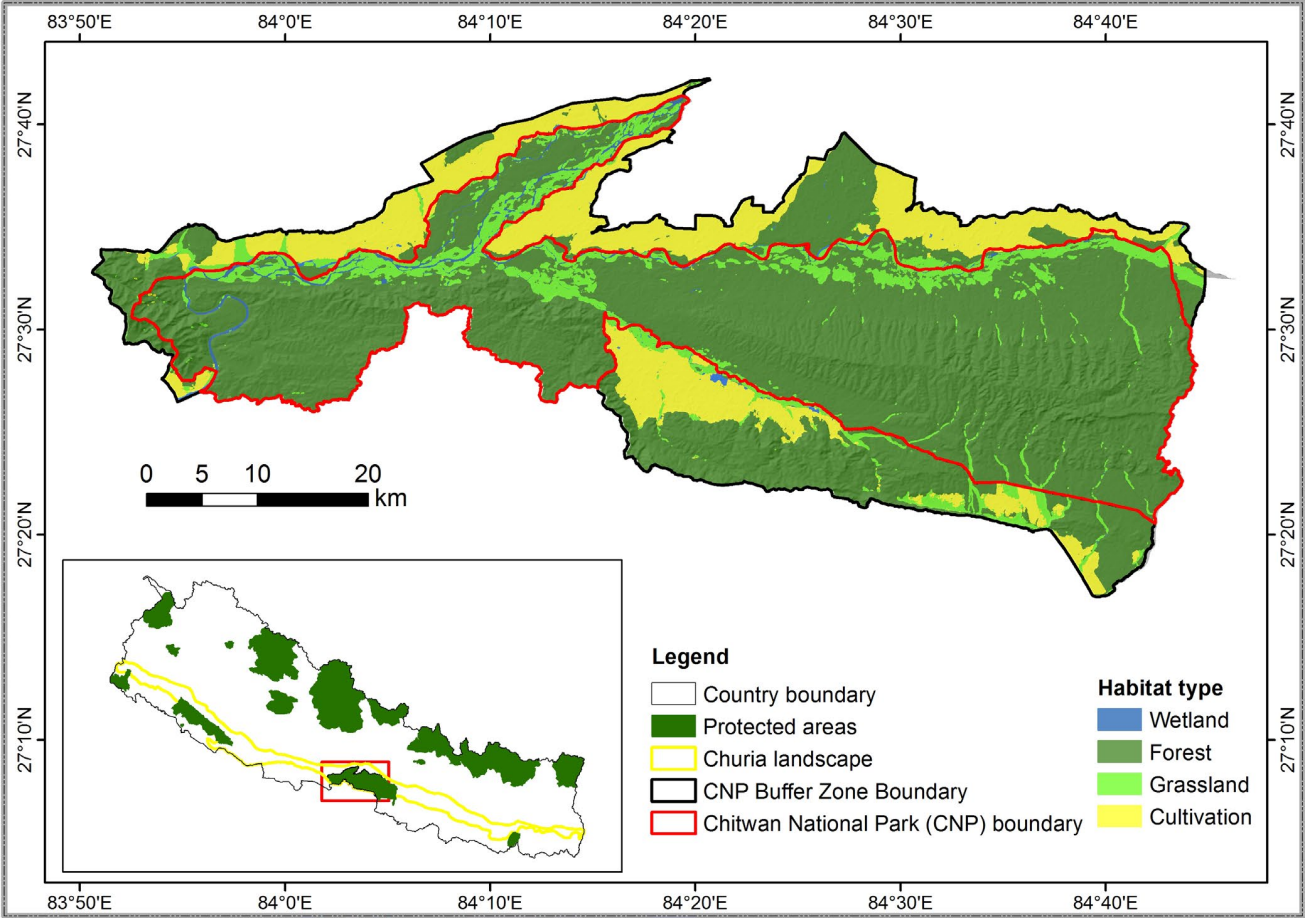
Study area map showing location and land cover pattern of Chitwan national park

CNP, a UNESCO world heritage site, was the first area in Nepal to receive protected status and covers 953 sq. km^2^ (Figure 2). The park is located in the south‐central part of Nepal along the floodplains of the Rapti, Reu, and Narayani rivers. The major vegetation cover consists of deciduous sal (*Shorea robusta*) forest (70%) followed by grassland (10%), riverine forest (7%), mixed forest (7%), and wetlands (4%). The successional gradient of the park is formed of 10 grassland and 3 forest associations (Lehmkuhl, 1999). Temperatures reach a maximum of 38°C during the summer and drop to a minimum of 6°C in winter. The average annual rainfall in the area is 2400 mm, most of which occurs during the summer monsoon. The matrix of different habitat conditions and climates makes this area a biodiversity hotspot. CNP harbors the largest populations of rhinos, tigers, sloth bears, and many other threatened flora and fauna in Nepal. The park is also a part of the Terai‐Duar savanna and Grasslands ecoregion, which is listed among the 200 most important areas globally (Dinerstein et al., 2017). Its resources are also of great importance to the livelihood of local people who depend strongly on forest resources for farming and livestock (Stræde & Treue, 2006). Local people are allowed to enter the core area of the park for approximately 2 weeks annually to collect grass, but the pressure for illegal access to park resources persists throughout the year (Sharma & Shaw, 1993; Stræde & Helles, 2000). The 750‐km^2^ area surrounding the park is delineated as a buffer zone. The buffer zone provides an extended habitat for wildlife and forest products for local communities, and also serves as an important area for eco‐tourism activities. Although poaching has not been excessive in recent years, human–wildlife conflicts are frequent in and around the park (Acharya et al., 2016; Lamichhane et al., 2018; Silwal et al., 2017). Furthermore, the impacts of global climate change on the local flora and fauna are predicted to intensify (Thapa et al., 2015).

### 2.2 Study design and field methods

We laid grids of 4 × 4 km over a map of the study area using QGIS 3.16. With a random starting position, we surveyed the grids in a checkboard pattern, sampling every other gird at a systematic spacing of 4 km. This checkerboard sampling design minimized autocorrelation between sampling grids, facilitated the concentration of survey efforts, ensured an even coverage of the large and hostile study area, and was suitable for studying medium‐to‐large mammals with relative ease. The same sampling method has been used to study elephants (Thapa et al., 2019), tigers (Thapa & Kelly, 2017), and four‐horned antelope (Krishna et al., 2008). This method yielded a total of 45 grids which covered 720 km^2^ (43% coverage of the park and buffer area). The grid size was comparable to the home range of sloth bears, which is estimated to be 9 and 14 km^2^ for male and female sloth bears, respectively (Joshi et al., 1995). We conducted sign surveys within the 45 grids, with a sampling effort of 4 km in each grid. We searched for sloth bear signs along a 4‐km‐long random walking trail that was divided into 20 continuous segments of 200 m. We identified grids on the ground, randomly selected a starting point in the first segment, and navigated within the grids using a handheld GPS. Within these segments, we collected sloth bear detection/non‐detection data and associated ecological, landscape, and anthropogenic variables. Detection of signs and covariates detected in a segment was recorded as “1”, otherwise “0”. If sampling could not proceed due to logistic reasons, or the area was outside park jurisdiction or under intense human use, the segment was treated as a missing observation. To standardize the detection process, avoid biases that may arise from the duplication, misidentification, and decay of signs, and adhere to the closure assumption in occupancy studies, we only included the first encounter of fresh sloth bear signs, that is, direct sightings, footprints, and scat along sample trails (Karanth et al., 2011; Morin et al., 2016; Putman, 1984; Rota et al., 2009). Field surveys were carried out between March and June of 2020. Sloth bears and Himalayan black bears are sympatric in the landscape further west of our study area particularly in the outer Himalaya, and the intervening valleys in Uttarakhand (India) and possibly in Bardiya National Park (Nepal) (Kadariya et al., 2018; Pigeon et al., 2018; Seidensticker et al., 2011; Yadav et al., 2017). However, Himalayan black bears have not been recorded in the present study area (Jnawali et al., 2011; Subedi, Bhattarai, et al., 2021; Subedi, Lamichhane, et al., 2021). The field team involved trained wildlife technicians who were able to unambiguously identify signs of bear presence.

### 2.3 Covariate selection

We selected a mix of six plausible remotely sensed and ground‐based variables that reflected the characteristics of the landscape, habitat conditions, and persistent anthropogenic pressures, as well as the availability of major food resources, based on a review of the available literature. For a small study area with a few sample sites, the model loses its power of explanation and the number of unwanted errors increases as the number of variables is increased in the model. It is generally advised to use 1 variable per 10 sites in an occupancy model. Thus, following the principles of parsimony, we included three site covariates and three sample covariates (Table 1). We selected termites, fruits, and disturbance as sample covariates and measured them in the field. Termites and fruits were selected as variables because they represent the dominant food resource for sloth bears (Bargali et al., 2012; Dharaiya et al., 2016; Joshi et al., 1997; Khanal & Thapa, 2014; Sukhadiya et al., 2013). We, therefore, predicted that the presence of termites and fruit trees would have a positive influence on bear detection and occupancy. In each segment, we recorded the presence/absence of termite mounds and fruit plants that were frequently consumed by sloth bears during the dry season in our study area (Khanal & Thapa, 2014). These variables were quantified at the grid level as the proportion of replicate segments in which they were present. We did not measure the absolute density of active or dormant termite mounds, and fruit‐bearing trees because of technical‐logistic limitations. Sloth bears have been reported to avoid human and livestock disturbances (Babu et al., 2015; Puri et al., 2015), but they have also been reported from human‐dominated landscapes with degraded habitats (Bargali et al., 2012). We combined human disturbance, livestock disturbance, and fire in our search trails as a measure of disturbance. A single disturbance score was prepared by taking the average value across segments. Sloth bears are thought to prefer relatively dry, rugged, and forested habitats (Puri et al., 2015; Srivathsa et al., 2018). We extracted the enhanced vegetation index (EVI) from Landsat 8 satellite data as a measure of vegetation productivity. We computed the topographic ruggedness index using the SRTM digital elevation model (Riley et al., 1999). In Nepal, it has been reported that sloth bears move to grasslands during the dry season and prefer to remain in forests during the wet season (Joshi et al., 1995). We extracted the tree cover data prepared by Hansen et al. (2013) using QGIS 3.16 as a proxy of habitat condition, with a higher cover indicating a forested habitat and a lower cover indicating a grassland habitat. All site covariates were first checked for collinearity. The results showed that none of the covariates were significantly correlated (Pearson's |*r*| = <0.5). We scaled and normalized all site covariates before running occupancy models (Krishna et al., 2008; Panthi et al., 2017). Based on the literature on sloth bear ecology, we hypothesized that sloth bear occupancy would increase with the increasing presence of termites and fruits and in dry, forested, and heterogeneous habitats.

**TABLE 1:** Description of covariates and the hypothesized response in occupancy (ψ) and detection (p) of sloth bears. “+” signifies a positive effect on the response variable, “−’ signifies a negative effect on the response variable

Covariate	Description	ψ	p	References
Enhanced vegetation index (EVI)	The EVI is similar to the normalized difference vegetation index but with a correction for some atmospheric conditions and canopy background noise, and is more sensitive in areas with dense vegetation cover. The EVI was derived from Landsat 8 thematic mapper imagery. A high EVI indicates moist and more productive areas, while a low EVI indicates drier areas	−	−	Sloth bears prefer relatively dry habitats and areas with a high vegetation productivity negatively influence sloth bear occupancy (Puri et al., 2015; Seidensticker et al., 2011)
Tree cover (Tcov)	Tcov was derived from data prepared by Hansen et al. (2013) and downloaded from the Global Forest Change website. A high Tcov indicates forested habitat, while a low Tcov indicates relatively open lowland habitats, such as grasslands	+	+	Sloth bears have been reported in a wide range of habitats, mostly forests, with some seasonal variation depending on the availability of food resources (Dharaiya et al., 2016; Joshi et al., 1995).
Terrain ruggedness index (TRI)	The TRI was computed using the Shuttle Radar Topography Mission digital elevation model (Riley et al., 1999) in QGIS 3.16. High coefficient of variation values in TRI indicated a large heterogeneity in terrain	+	+	The rugged terrain provides sloth bears with resting and denning refuge and positively influences sloth bear occupancy (Akhtar et al., 2007; Puri et al., 2015; Yoganand, 2005)
Disturbance (Dist)	Presence/absence scores of humans, livestock, and fire were recorded in the field and pooled to obtain an average Dist score as a surrogate for human impact. A high Dist score indicated more human impact, while a low score indicated less human impact on the habitat	−	−	Sloth bears largely prefer habitats away from human disturbance (Babu et al., 2015; Baskaran et al., 2015; Das et al., 2014; Joshi et al., 1999; Puri et al., 2015)
Fruit (Frut)	The presence/absence of fruit plants most frequently consumed during the dry season in Chitwan (Khanal & Thapa, 2014) was pooled to obtain an average fruit score for each grid and recorded as the proportion of trail segments with the presence of fruit trees	+	+	Termites and fruits are the major components of sloth bear diet that influence its distribution and habitat use (Das et al., 2014; Dharaiya et al., 2016; Joshi et al., 1997; Khanal & Thapa, 2014; Laurie & Seidensticker, 1977)
Termite (Term)	The presence/absence of termites was recorded in the field and a single score for each grid was obtained by quantifying it as the proportion of trail segments with the presence of termite mounds.	+	+	

### 2.4 Occupancy estimation and modeling the effects of covariates

Spatial replication can serve as a good surrogate for temporal replication in occupancy studies of sloth bears if an appropriate modeling framework is used to account for the particular sampling process (Srivathsa et al., 2018). Standard occupancy models (MacKenzie et al., 2002) that assume independence between replicates to separate non‐detection from absence were not suitable for our single‐season dataset collected along adjacent trail segments. However, Hines et al. (2010) modeling approach accounts for such spatial dependence between replicates. This approach does not assume that in an occupied grid all spatial replicates are occupied but rather estimates two additional parameters, *θ* ^o^ and *θ* ^1^, representing the replicate‐level presence of the species, which is conditional on signs being absent or present in the previous replicate, respectively. We compared a standard single‐season occupancy model and correlated detection model to identify an appropriate model for our data. We compared these models based on the Akaike information criterion (AIC) and selected the model with the lowest AIC score (Burnham & Anderson, 1998). This comparison indicated the spatial dependencies in sign detection in our replicate segments, with a lower AIC value (better model performance) for the spatial correlation model than the standard occupancy model (Table 2). We, therefore, used a spatial correlation model (Hines et al., 2010) for further analysis. We ran a single‐species single‐season occupancy analysis using a maximum likelihood‐based approach in the PRESENCE 2.12.31 software (Hines, 2006). While modeling covariate effects, we could not ignore the possibility that covariates influencing sloth bear presence would also affect sloth bear detectability due to occupancy–abundance relationships. We followed a two‐step process to estimate the probability of detection (p) and probability of bear occurrence (ψ). First, we modeled detection by keeping a global covariate structure for the occupancy model as ψ (Global). This global model included all six covariates (i.e., termites, fruits, disturbance, tree cover, terrain heterogeneity, and vegetation productivity) that could influence the probability of bear occurrence. We modeled different combinations of the detectability covariates for ψ (Global) and selected the best model based on the minimum AIC. In the second step, we modeled the probability of occupancy (ψ) by keeping the top detection model from the previous step as a constant structure in the detection model (Doherty et al., 2012; Panthi et al., 2017; Srivathsa et al., 2018). We modeled the covariates stepwise beginning with the univariate model structure. If the addition of covariates improved the model fit, then it was retained to be combined with the other covariates in multivariate models. The candidate model set included either the single or additive effects of two or more covariates to investigate the influence of covariates on occurrence. Model fit was assessed using the parametric bootstrap procedure (MacKenzie & Bailey, 2004). The covariate models were compared and ranked using an information theoretic approach, relying on the AIC for testing relative model fits. Due to the inherent advantage of model averaging (Burnham & Anderson, 1998), the final occupancy estimates and associated standard error were averaged across the model set. To infer the relative influence of covariates on occurrence, we used the estimated *β*‐coefficients of the model containing the particular covariate.

**TABLE 2:** Summary of the model selection process for factors influencing detection probability of Sloth bear

Model	AIC	ΔAIC	W*_i_*	ML	*K*
ψ (Global),th0(),th1(), p(Term + EVI + TRI),th0pi()	468.46	0.00	0.37	1.00	14.00
ψ (Global),th0(),th1(), p(Term+EVI),th0pi()	468.59	0.13	0.35	0.94	13.00
ψ (Global),th0(),th1(), p(Term),th0pi()	470.71	2.25	0.12	0.32	12.00
ψ (Global),th0(),th1(), p(EVI),th0pi()	472.83	4.37	0.04	0.11	12.00
ψ (Global),th0(),th1(), p(.),th0pi()	472.99	4.53	0.04	0.10	11.00
ψ (Global),th0(),th1(), p(TRI),th0pi()	473.93	5.47	0.02	0.06	12.00
ψ (Global),th0(),th1(), p(Dist),th0pi()	474.35	5.89	0.02	0.05	12.00
ψ (Global),th0(),th1(), p(Frut),th0pi()	474.39	5.93	0.02	0.05	12.00
ψ (Global),th0(),th1(), p(Tcov),th0pi()	474.94	6.48	0.01	0.04	12.00

Abbreviations: AIC, Akaike's information criterion; Dist, Disturbance; EVI, Enhanced Vegetation Index; Frut‐, Fruit; *K*, Number of parameters estimated by the model; ML, Model likelihood; p, probability of detection; Term, Termite; TRI, Terrain Ruggedness Index; W*_i_*, AIC model weight; ΔAIC, the difference in the AIC values between each model and the model with the lowest AIC; ψ, probability of occupancy.

## 3 RESULTS

We first compared the standard occupancy model (MacKenzie et al., 2002) and spatial correlation model (Hines et al., 2010). The model developed by Hines et al. (2010), which accounted for spatial dependencies in sign detection along our replicates, received more support from the data compared to MacKenzie et al. (2002) modeling approach (ΔAIC of ψ (.),p(.) =16.7, relative to ψ(.) th0(.),th1(.),p(.),th0pi(.)). We then fitted models with different combinations of the detectability (p) covariates, keeping the global covariate structure for occupancy ψ (Global) (Table 2). All candidate models had some level of support based on the AIC values and corresponding model weights, and no single model received unequivocal support from the data. We estimated detectability from the best performing model with the lowest AIC value (*p* = .25 ± 0.05_SE,_ W*_i_* = 0.37). This detectability model suggested that sloth bear detection increased with an increase in the presence of termite mounds (*β*_Term_ = 0.75 ± 0.34_SE_), drier habitats (*β*_EVI_ = −0.46 ± 0.19_SE_), and non‐heterogeneous terrain (*β*_TRI_ = −0.36 ± 0.25_SE_). We used this detectability model in subsequent analyses to model occupancy probability. We fitted occupancy models in a stepwise additive process (Table 3). We also ran all covariate structures for modeling occupancy using the next best detection model (Term + EVI, ΔAIC = 0.13, W*_i_* = 0.35) as it also received similar support from the data. Among the set of candidate models, the model including termites (*β*_Term_ = 1.08 ± 0.60_SE,_ W*_i_* = 0.76) was the best occupancy model. Because of the inherent advantages of model averaging (Burnham & Anderson, 1998), we averaged across all models to estimate the probability of sloth bear occupancy at ψ = 0.69 ± 0.24_SE_. The model‐specific *β*‐coefficient value from the occupancy models for termites (*β*_Term_ = 1.08 ± 0.60_SE_), fruit (*β*_Frut_ = 0.10 ± 0.14_SE_), and terrain heterogeneity (*β*_TRI_ = 0.50 ± 0.29_SE_) indicated their positive influence on sloth bear occupancy, whereas the negative *β*‐coefficients for disturbance (*β*_Dist_ = −0.26 ± 0.16_SE_), tree cover (*β*_Tcov_ = −0.14 ± 0.14_SE_), and vegetation productivity (*β*_EVI_ = −0.31 ± 0.23_SE_) indicated their negative associations with sloth bear habitat occupancy (Table 4).

**TABLE 3:** Summary of the model selection process for factors influencing Sloth bear occupancy

Model	AIC	ΔAIC	W*_i_*	ML	*K*
ψ (Term),th0(),th1(), p(Term + EV + TRI),th0pi()	465.85	0	0.76	1	9
ψ (Dist),th0(),th1(), p(Term + EVI + TRI),th0pi()	470.95	5.10	0.06	0.08	9
*Ψ(.),th0(),th1(), p(Term* + *EVI),th0pi()*	471.63	5.78	0.04	0.06	7
ψ (EVI),th0(),th1(), p(Term + EV + TRI),th0pi()	471.93	6.08	0.04	0.05	9
ψ (.),th0(),th1(), p(Term + EV + TRI),th0pi()	472.00	6.15	0.04	0.05	8
ψ (TRI),th0(),th1(), p(Term + EV + TRI),th0pi()	472.03	6.18	0.03	0.05	9
ψ (Tcov),th0(),th1(), p(Term + EV + TRI),th0pi()	473.04	7.19	0.02	0.03	9
ψ (Frut),th0(),th1(), p(Term + EV + TRI),th0pi()	473.44	7.59	0.02	0.02	9

Note

Second best detection model *Ψ(*.*)*, *th0()*, *th1()*, *p(Term*+*EVI)*, *th0pi ()* included in occupancy modeling along with the best detection model (Term+EVI+TRI).

Abbreviations: AIC, Akaike's information criterion; Dist, Disturbance; EVI, Enhanced Vegetation Index; Frut, Fruit; *K*, Number of parameters estimated by the model; ML, Model likelihood; p, probability of detection; Tcov, Tree Cover; Term, Termite; TRI, Terrain Ruggedness Index; W*_i_*, AIC model weight; ΔAIC, the difference in the AIC values between each model and the model with the lowest AIC; ψ, probability of occupancy.

**TABLE 4:** Comparison of the relative strength of covariate influence on sloth bear occupancy and detection

Covariates	Occupancy			Detection		
	*β* (SE)	LCI	UCI	*β* (SE)	LCI	UCI
Termite (Term)	1.08 (0.60)	−0.09	2.25	0.75 (0.34)	0.09	1.41
Fruit (Frut)	0.10 (0.14)	−0.17	0.38	0.27 (0.35)	−0.42	0.96
Disturbance (Dist)	−0.26 (0.16)	−0.56	0.05	0.69 (0.87)	−1.01	2.39
Tree cover (Tcov)	−0.14 (0.14)	−0.42	0.14	0.04 (0.16)	−0.27	0.35
Terrain ruggedness (TRI)	0.50 (0.29)	−0.08	1.07	−0.30 (0.31)	−0.91	0.31
Vegetation productivity (EVI)	−0.31 (0.23)	−0.76	0.13	−0.35 (0.20)	−0.74	0.04

Abbreviations: LCI, Lower confidence interval; UCI, Upper confidence interval; *β* (SE), Beta coefficient (standard error).

## 4 DISCUSSION

### 4.1 Occupancy and detection

This study provided the first occupancy estimate for sloth bears from CNP, Nepal. Their signs were detected in 21 of the 45 grids sampled, giving a naive occupancy of 46%. By explicitly incorporating the imperfect detection of animals into the occupancy estimate, the proportion of area occupied by sloth bears in CNP substantially increased to 69% with a model‐averaged detection probability of 0.25. Hines et al. (2010) approach estimates the probability of detecting the species in a spatial replicate, given its presence in the site as well as its presence in the replicate, while the MacKenzie et al. (2002) approach calculates the probability of detecting the species in a site given its presence in the site. Because of this additional conditioning on presence in the spatial replicate, estimates from Hines et al. (2010) tend to be higher than from the MacKenzie et al. (2002) approach. The large increase in habitat occupancy over the naive estimate highlights the importance of considering the imperfect detection using an appropriate occupancy approach when studying sloth bears.

**FIGURE 3:**
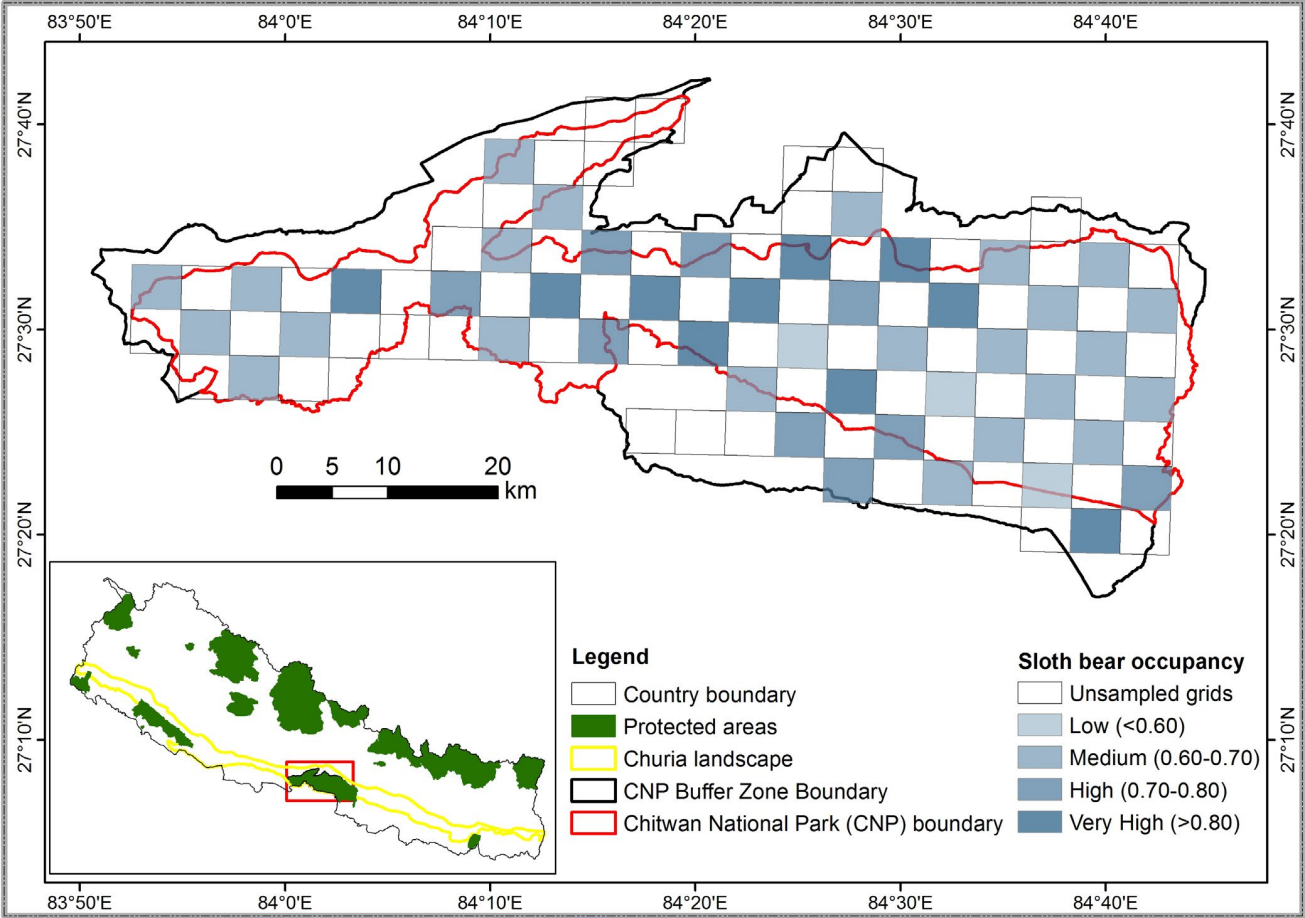
Study area map showing the probability of sloth bear occupancy in Chitwan national park

Estimates of habitat occupancy by sloth bears and effects of covariates vary across studies within its distribution range. Discrepancies in the landscape composition, scale of the study, nature of data, and methods used may preclude direct comparisons of occupancy estimates and the effect of covariates across studies in different landscapes. In India, habitat occupancy was estimated at 57% in Bhadra Wildlife Sanctuary (Srivathsa et al., 2018), 61% in the Malenad region (Puri et al., 2015), 79% in different regions of northeastern Karnataka (Das et al., 2014), and 83% in the Mudumalai Tiger Reserve (Ramesh et al., 2012). Most of the reported studies of sloth bear occupancy in India are from the Western Ghats, which has large blocks of contiguous forest cover and a diversity of habitat conditions, with semi‐evergreen, tropical moist, dry deciduous, thorny forest, and scrub landscapes interspersed with agricultural areas and rocky outcrops, while our study area was relatively homogenous with small grasslands patches interspersed in a deciduous forest habitat. Sloth bears have a small home range (9–14 km^2^) in CNP (Joshi et al., 1995) compared to Central India (12–85 km^2^) (Yoganand, 2005), indicating a possible availability of resource‐rich habitat for sloth bears in CNP. In the unprotected Trijuga forest area of Udaypur and Saptari districts, approximately 200km east of CNP, the probability of habitat use was estimated much lower at 43% (Pokharel et al., 2022). Variation in patterns of habitat use by sloth bears is a characteristic of most bear species; bears exhibit high diversity, complexity, and adaptability in their use of habitat mostly depending on the diversity and quantity of foods, and habitat conditions providing shelter and safety from human and non‐human predators like tigers (Garshelis, 2022). Species tend to exhibit occupancy–abundance relationships (Gaston et al., 2000; Zuckerberg et al., 2009), particularly in small and homogenous areas (Hui et al., 2009). This indicates that sloth bears are fairly abundant and have a wide distribution throughout the park. Relatively high‐occupancy areas (psi > 0.70) were located in the central‐north area of the park (Figure 3). Both Laurie and Seidensticker (1977), as well as Garshelis et al. (1999), recognized that there was an uneven distribution of sloth bears with a high density in the alluvial floodplains and a relatively lower density in the rest of the park, which is dominated by upland sal forest.

### 4.2 Influence of covariates

We assessed the importance of different covariates based on the magnitude of the estimated *β*‐coefficients. The summed AIC weight from the models could not be used to determine the relative importance of covariates because our model set was not balanced with respect to the representation of covariates across the models. Because we scaled and normalized occupancy covariates, their *β*‐coefficient represented the change in logit (ψ) for 1 standard deviation change in the covariate. The model‐specific *β*‐coefficient value from the occupancy models indicated that termites, fruit, and terrain heterogeneity had positive influences on sloth bear occupancy, whereas disturbance, tree cover, and vegetation productivity had negative associations with sloth bear habitat occupancy.

The food resources of sloth bears, particularly termites, had a relatively strong influence on sloth bear occupancy. This was expected because sloth bears are opportunistic omnivores that are specialized for a myrmecophagous diet (Joshi et al., 1997, 1999). Studies of their feeding ecology have shown that termites are the most frequent dietary item throughout the year, while fruit consumption is dependent on seasonal availability (Bargali et al., 2004; Palei et al., 2014, 2020; Ramesh et al., 2012; Rather et al., 2020; Yoganand, 2005). In Chitwan, fruits are available for a short period from April to August, while termites tend to increasingly dominate the sloth bear's diet. Their presence was detected in 52% of scats in the 1970s (Laurie & Seidensticker, 1977), 81% during the 1990s (Joshi et al., 1997), and 92% in the 2010s (Khanal & Thapa, 2014). The presence of sloth bears was negatively associated with tree cover, indicating a preference for open grassland habitats. Forest and grassland associations provide a habitat mosaic and are a key determinant of mammalian abundance in CNP (Bhattarai & Kindlmann, 2012; Lehmkuhl, 1999). Another study in CNP suggested that an abundant food supply during the dry season would prompt the movement of sloth bears from dense sal forests to open grassland areas (Joshi et al., 1995). Despite the higher density of termite mounds in sal forest compared to mixed or open habitats (Axelsson & Andersson., 2012; Chakraborty & Singh, 2020), based on their diggings there was more evidence of sloth bears in grassland habitats during the dry season (Garshelis et al., 1999). During the dry season, the soil in upland sal forest habitats becomes stiff (Malla & Karki, 2016). Termites excavate deeper into the ground to seek moisture (Ahmed & Pradhan, 2018; Sen‐Sarma, 1974). Obtaining termites from stiff mounds becomes difficult in forests compared to grassland habitats where the soil is relatively loose, making it less likely that sloth bears will dig into mounds and underground colonies of termites and ants (Garshelis et al., 1999; Joshi et al., 1995, 1997). It seems likely that the distribution of sloth bears in CNP is seasonal, and depends on the seasonal variation of food sources. Therefore, our results may have differed if multi‐season sampling were used. There may also be negative associations with tree cover because our sampling design may have resulted in higher coverage of peripheral areas that consist of grasslands, riverine forests, and buffer zones, while most of the dense forest lies in the core of the park.

Habitat occupancy was negatively associated with disturbance, indicating that sloth bears avoid disturbed and degraded habitats. Human activities are the predominant factors that determine areas of occupancy within the sloth bear range (Seidensticker et al., 2011). Multiple factors, such as individual behavior and evolutionary history, as well as the frequency, duration, and scale of disturbance events, influence species occupancy (Graham et al., 2021; Iwasaki & Noda, 2018; Sousa, 1984). In relatively intact landscapes, such as the Western Ghats in India, sloth bears have been shown to avoid disturbance (Babu et al., 2015; Das et al., 2014; Puri et al., 2015), while in human‐dominated landscapes they have been reported to tolerate some degree of disturbance (Bargali et al., 2012), often consuming cultivated crops (Palei et al., 2020) and human food waste (Prajapati et al., 2021), and causing conflicts with humans (Debata et al., 2017; Dhamorikar et al., 2017). Human–sloth bear conflict is common throughout the year in CNP, suggesting that sloth bears perceive humans as a threat (Acharya et al., 2016; Lamichhane et al., 2018; Silwal et al., 2017). Previous reports of sloth bears from degraded forests were likely because the study was conducted in an area of degraded forests and should not be taken as the norm in terms of sloth bear ecology (Rather et al., 2021) but rather as the manifestation of a high nexus between sloth bears and humans in the landscape. Sloth bears might use disturbed habitats in moderation for food, water, and shelter. In a few instances, we sighted sloth bears and their signs in fissures and crevices along the forest, and along river paths used by humans. A rugged terrain provides sloth bears with resting and denning sites (Akhtar et al., 2007; Bargali et al., 2012; Baskaran et al., 2015), as well as cover to hide their cubs from potential predators, such as tigers. Terrain heterogeneity was positively related to the habitat occupancy of sloth bears. Enhanced vegetation productivity was negatively associated with sloth bear occupancy, suggesting a preference for dry habitats. A similar preference for heterogeneous and dry habitats was reported for sloth bears in India (Puri et al., 2015).

The 95% confidence interval of β‐coefficients for the occupancy covariates overlapped zero indicating weak statistical support for the magnitude of influence of variables. Our study results were limited by the small sample size and single‐season sampling. The scale of our study, use of grid size comparable to the home range of sloth bears in the study area, and adoption of a checkerboard sampling design for wider coverage, and efficient sampling amid logistic challenges resulted in a relatively small sample size. While few studies from small areas report estimates based on small sample size (Lamichhane et al., 2020; Thapa et al., 2017), others use smaller sampling units (Babu et al., 2015; Das et al., 2014;) or use occupancy estimates as the intensity of habitat use (Thapa & Kelly, 2017; Thapa et al., 2019). Sampling units should be larger than the estimated home range of species to measure the true estimate of occupancy (Karanth et al., 2011; MacKenzie & Royle, 2005). It is suggested that for a rare species, it is more efficient to survey more sampling units less intensively, while for a common species fewer sampling units should be surveyed more intensively (MacKenzie & Royle, 2005). Limited sample and poor detectability make it difficult to disentangle the occupancy and detection process, and fully retrieve species–environment relationships (Guillera‐Arroita et al., 2014; MacKenzie & Bailey, 2004). Furthermore, the use of a step‐wise modeling approach may increase the risk of the possible overfitting of data that might not hold up to generalizations. Cautious application of occupancy methods by sampling in more sites with larger replication may produce more precise and robust inferences. The additional quantified measurement of active termite mounds, underground colonies of termites and ants, fruit‐bearing trees, and disturbance intensity may be required to provide a deeper understanding of the ecological interactions and behavioral responses of the sloth bear. The results would likely change if standard multi‐season sampling were adopted. Nevertheless, our findings fill an important information gap on sloth bears in Nepal, while many contemporary wildlife research and conservation programs are focused on large and charismatic species.

## 5 IMPLICATIONS FOR CONSERVATION

Our results indicated that sloth bears are widespread but elusive in CNP. The probability of their detection and occupancy was mostly influenced by the presence of termites and a range of ecological, landscape, and anthropogenic variables. Landscape features such as ruggedness change over decades, however, habitat variables such as tree cover, vegetation productivity, and the availability of fruits and insects change over short time periods. While generalist species may adapt to such changes, the specific feeding and habitat requirements of sloth bears make this species more vulnerable. Habitat changes can have consequences for the long‐term survival of species if they contribute to the loss of genetic diversity and population decline (Dutta et al., 2015; Murphy et al., 2017; Thatte et al., 2020). Studies have shown a decline in sympatric carnivores where conservation is focused on the revival of a single species such as tigers (Jhala et al., 2020; Li et al., 2020). Tigers and sloth bears co‐occur in Nepal, where the former's population has almost doubled since 2009 (DNPWC & DFSC, 2018). Direct threats to sloth bear populations through predation by tigers (Joshi et al., 1999) might be low, but indirect consequences of habitat alteration due to tiger‐focused management can be expected. Grassland habitat in the park is shrinking due to the proliferation of shrub, woody vegetation, and invasive alien plants, which is already impacting grassland‐dependent species (Murphy et al., 2013; Subedi et al., 2017). The intactness of the habitat and the ability of species to survive and reproduce is further challenged by anthropogenic pressure, which is exacerbated by the increasing impacts of climate change (Pant et al., 2020). Therefore, the fate of this unique ursid not only relies on how it responds to the changing availability of insects, fruits, and habitat but also on how park managers respond through management actions or inactions. Our study provides general guidance to parks and wildlife conservation authorities toward a departure from incidental conservation to active management of the sloth bear population. Management actions should be geared toward the creation of suitable habitat that enables sloth bears to access their foods throughout the year and successfully reproduce. Its unique characteristics and ecological importance make the sloth bear a potential umbrella species (Puri et al., 2015; Ratnayeke & Manen, 2012). Our results and the recent reports of sloth bears outside the protected area along the Churia landscape (Pokharel et al., 2022; Subedi, Bhattarai, et al., 2021; Subedi, Lamichhane, et al., 2021) hint to such a possibility in Nepal. However, the lack of rigorous assessments within and outside CNP remains a major barrier to fully understanding its abundance, ecological interactions, and conservation importance. Our findings are a valuable baseline for future actions and strategies aimed at sloth bear conservation and management in Nepal.

## CONFLICT OF INTEREST

No conflict of interest.

## AUTHOR CONTRIBUTIONS

**Rajan Prasad Paudel:** Conceptualization (equal); Data curation (lead); Formal analysis (lead); Funding acquisition (equal); Writing – original draft (lead); Writing – review & editing (equal). **Rabin Kadariya:** Conceptualization (equal); Formal analysis (supporting); Funding acquisition (supporting); Writing – review & editing (supporting). **Babu Ram Lamichhane:** Formal analysis (supporting); Methodology (supporting); Writing – review & editing (supporting). **Naresh Subedi:** Conceptualization (equal); Supervision (equal). **Mariko Sashika:** Supervision (equal). **Michito Shimozuru:** Supervision (equal); Writing – review & editing (supporting). **Toshio Tsubota:** Funding acquisition (equal); Supervision (lead).

## CONSENT TO PARTICIPANTS

Not applicable.

## Data Availability

Data associated with this manuscript can be accessed at the Dyrad data repository (https://doi.org/10.5061/dryad.c59zw3r7s).
